# Moderate Hyperbilirubinemia Alters Neonatal Cardiorespiratory Control and Induces Inflammation in the Nucleus Tractus Solitarius

**DOI:** 10.3389/fphys.2016.00437

**Published:** 2016-09-30

**Authors:** Marie-Laure Specq, Mélisande Bourgoin-Heck, Nathalie Samson, François Corbin, Christian Gestreau, Maxime Richer, Hazim Kadhim, Jean-Paul Praud

**Affiliations:** ^1^Neonatal Respiratory Research Unit, Departments of Pediatrics and Pharmacology - Physiology, Université de SherbrookeSherbrooke, QC, Canada; ^2^Department of Biochemistry, Université de SherbrookeSherbrooke, QC, Canada; ^3^Aix-Marseille University, CNRS, CRN2MMarseille, France; ^4^Department of Pathology, Université de SherbrookeSherbrooke, QC, Canada; ^5^Neuropathology Unit and Reference Center for Neuro-Muscular Pathology, Brugmann University Hospital and Childrens' Hospital (CHU Brugmann - HUDERF), Université Libre de BruxellesBrussels, Belgium

**Keywords:** hyperbilirubinemia, prematurity, brain, pathophysiology, pulmonary and laryngeal chemoreflexes, hypoxia, nucleus tractus solitarius

## Abstract

Hyperbilirubinemia (HB) occurs in 90% of preterm newborns. Moderate HB can induce acute neurological disorders while severe HB has been linked to a higher incidence of apneas of prematurity. The present study aimed to test the hypothesis that even moderate HB disrupts cardiorespiratory control in preterm lambs. Two groups of preterm lambs (born 14 days prior to term), namely control (*n* = 6) and HB (*n* = 5), were studied. At day 5 of life, moderate HB (150–250 μmol/L) was induced during 17 h in the HB group after which cardiorespiratory control as well as laryngeal and pulmonary chemoreflexes were assessed during baseline recordings and during hypoxia. Recordings were repeated 72 h after HB induction, just before euthanasia. In addition, neuropathological studies were performed to investigate for cerebral bilirubin deposition as well as for signs of glial reactivity in brainstem structures involved in cardiorespiratory control. Results revealed that sustained and moderate HB: (i) decreased baseline respiratory rate and increased the time spent in apnea; (ii) blunted the cardiorespiratory inhibition normally observed during both laryngeal and pulmonary chemoreflexes; and (iii) increased heart rate in response to acute hypoxia. These acute physiological changes were concurrent with an activation of Alzheimer type II astrocytes throughout the brain, including the brainstem. Concomitantly, bilirubin deposits were observed in the leptomeninges, but not in brain parenchyma. While most cardiorespiratory alterations returned to normal 72 h after HB normalization, the expression of glial fibrillary acid protein (GFAP) and ionized calcium binding adaptor molecule 1 (Iba1) was still increased within the nucleus tractus solitarius. In conclusion, moderate and sustained HB in preterm lambs induced cardiorespiratory alterations, the latter of which were associated with neurohistopathological changes. These changes are indicative of an inflammatory response in the brainstem neuroanatomical substrates involved in cardiorespiratory control.

## Introduction

Hyperbilirubinemia (HB) is one of the most common problems encountered in neonates, particularly preterms (Watchko, 2009). Neurotoxicity is the major consequence of neonatal HB and includes a host of neurological dysfunctions such as somnolence, abnormal muscle tone, feeding difficulties, and auditory dysfunction. Kernicterus, the most severe form of neurotoxicity, is responsible for long-term neurological sequelae, and although now very rare, milder forms of acute bilirubin-induced neurological dysfunction still remain frequent (Johnson and Bhutani, 2001; Mazeiras et al., 2012). A few studies have also suggested an association between HB and apneas and/or bradycardias in newborn infants (Johnson et al., 2009; Amin et al., 2014; Amin and Wang, 2015). Furthermore, a study in rat pups provided evidence that acute HB influences respiratory control; however, the degree of HB was much higher and more transient than levels clinically encountered in neonates (Mesner et al., 2008). More recently, we showed a decreased efficiency in bottle-feeding and an impaired swallowing-breathing coordination in a novel experimental model of sustained and moderate HB in preterm lambs (Bourgoin-Heck et al., 2015). Overall, the above studies suggest that HB induces respiratory alterations in the neonate. The main objective of the present study was to test the hypothesis that moderate HB, at a level similar to that commonly observed in newborn infants, induces acute and delayed alterations in cardiorespiratory control. In addition, we aimed to further elucidate the mechanisms involved in these alterations by assessing whether neurohistopathological alterations are present in the region of the nucleus tractus solitarius, which is devoted to central processing of cardiorespiratory afferent inputs (Kubin et al., 2006).

## Materials and methods

The study protocol was approved by the Ethics Committee for Animal Care and Experimentation of the Université de Sherbrooke (protocol # 260–10).

### Preterm lamb model of moderate hyperbilirubinemia

Sixteen preterm lambs were born vaginally on gestational day 133 (term = 147 days) as previously described (Boudaa et al., 2013). Results on the effects of HB on bottle-feeding efficiency and nutritive swallowing-breathing coordination obtained in the same preterm lambs have previously been published (Bourgoin-Heck et al., 2015). Premature labor was induced by mifepristone (8 mg/kg), preceded by betamethasone (12 mg × 2). A unique preterm lamb model for moderate HB was designed for the purposes of the study (Bourgoin-Heck et al., 2015). Briefly at birth, lambs were randomly assigned to either the HB group or control group. On postnatal day 5, HB was induced by an intravenous infusion of 20 mg/kg of the bilirubin solution, stabilized with albumin and diluted in Ringer's Lactate buffer (Hansen et al., 1992; Mesner et al., 2008; Bourgoin-Heck et al., 2015), whereas control lambs received a bilirubin-free solution. Bilirubinemia was maintained between 150 and 250 μmol/L for a period of 17 h.

Neurological consequences of HB were characterized based on the “Lamb Acute Bilirubin Encephalopathy score” as well as on consistent electroencephalogram changes, as previously described (Bourgoin-Heck et al., 2015). Accordingly, the presence of acute bilirubin encephalopathy was assessed according to published guidelines for the detection of HB in newborns (American Academy of Pediatrics Subcommittee on Hyperbilirubinemia, 2004). Clinical examinations (consciousness, muscle tone, and audition) were repeated every 2 h throughout bilirubin infusion, then twice daily until the end of the experiment. Detailed results of the LABE score are presented for every lamb in Table 1 of the digital supplement of Bourgoin-Heck et al. (2015). In addition to the clinical detection of acute bilirubin encephalopathy, an electroencephalogram (EEG) was recorded for 6 h during bilirubin infusion and again 6 h at D3, in particular for detecting alterations in sleep organization or epileptiform discharges in HB lambs.

Finally, daily monitoring included vital signs (heart rate, respiratory rate, pulse oximetry, rectal temperature), weight and blood samples for albumin, glucose, bilirubin, and arterial blood gases.

### Instrumentation and recording equipment

Animals were chronically instrumented on postnatal day 5 and thereafter, and included pairs of needle-electrodes inserted subcutaneously: (i) above the occipital cortex for electroencephalogram (EEG); (ii) near to the right eye socket for electrooculogram (EOG); and (iii) on both sides of the thorax for electrocardiogram (ECG). In addition, the following were implanted: (iv) catheters into both jugular veins (IV infusion and injection; blood sampling); (v) a 5 Fr naso-pharyngeal catheter with its tip positioned 0.5 cm above the posterior border of the soft palate [induction of laryngeal chemoreflexes (LCR)]; (vi) elastic bands around the thorax and abdomen for respiratory inductance plethysmography (respiratory movements; NIMS, Miami Beach FL, USA); (vii) a Masimo pulse oximeter sensor at the tail base [arterial oxygen hemoglobin saturation (SpO_2_)]. Lastly, in order to assess the response to hypoxia, a custom-built nasal mask molded to fit the lamb's muzzle (Samson et al., 2005) was connected to a pneumotachograph (flow transducer 47304A Hewlett-Packard, Hewlett-Packard Company, Palo-Alto, CA, USA) and a 2-way system which provided either medical air or a gas mixture of 10% O_2_ in air. Arterial blood gases were also measured in two lambs from each group during hypoxia from an arterial brachial catheter surgically inserted under local anesthesia, prior to recordings.

Leads from all sensors were connected to our custom-built radio telemetry transmitters (Samson et al., 2011). All signals were continuously recorded using AcqKnowledge software (version 4.1, Biopac Systems Canada Inc., Montreal, Qc, Canada) and the lambs were filmed using a web-cam.

### Design of the study

**Figure 1:**
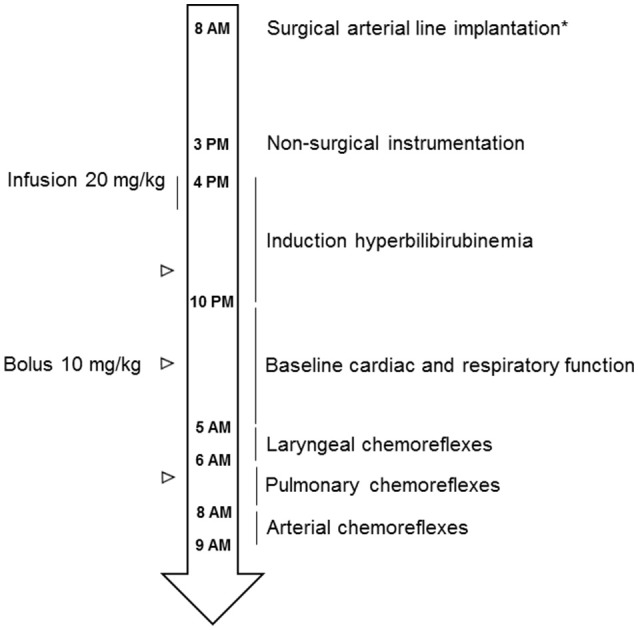
**Schematic drawing of the experimental design**. ^*^Surgical arterial line implantation was performed in only 4 lambs (2 control and 2 HB lambs).

All preterm lambs were housed and cared for with their dams in our animal quarters until the experimental day. Polysomnographic recordings were first performed on postnatal day 5, named experimental day 0 (D0), to assess the immediate effects of moderate HB on baseline cardiac and respiratory activity, as well as on cardiac and respiratory responses to several chemical stimuli, including laryngeal and pulmonary chemoreflexes and hypoxia (see below and Figure 1). Recordings were repeated 72 h after HB induction (D3) to assess the delayed effects of moderate HB.

#### Baseline cardiac and respiratory function

States of alertness, ECG, respiratory movements, hemoglobin O_2_ saturation, and pulse rate were continuously recorded for a period of 7 h from 10:00 p.m. to 5:00 a.m., immediately after reaching the bilirubinemia target. During these polysomnographic recordings, the non-sedated lambs were placed in a Plexiglas chamber (1.2 × 1.2 × 1 m, corresponding to guidelines from the Canadian Council for Animal Care) in which they could move freely. After 3 h of recording, they were temporarily returned to their mothers in order to feed before being brought back to the Plexiglass chamber for the remainder of the recording session.

#### Cardiorespiratory responses to chemical challenges

##### Laryngeal chemoreflexes

Immediately following baseline cardiac and respiratory activity recordings, LCR were induced by two separate injections of 2 ml of hydrochloric acid (HCl, pH = 2, diluted in saline) through the naso-pharyngeal catheter during NREM sleep, after a 3 min baseline recording. Each lamb was given at least 30 min of recovery time between the two injections. Events such as agitation, cough, arousal and/or full awakening were noted by an observer present throughout the recordings, in addition to the continuous video recording.

##### Pulmonary chemoreflexes

Pulmonary chemoreflexes (Diaz et al., 1999) were induced during NREM sleep, 30 min after LCR assessment. Following a 3 min baseline recording, lambs were first given an IV bolus injection of 1 ml of vehicle (i.e., 10% Tween 80–10% ethanol—80% saline). After a five-min delay, two pulmonary chemoreflexes were induced at a 30 min interval by central IV injections of 25 μg/kg capsaicin (diluted in vehicle; Sigma Chemical, St-Louis, MO, USA).

##### Hypoxia

At the end of each experimental day, preterm lambs were exposed to 10% poïkilocapnic hypoxia. Following a 3 min baseline recording in normoxia (21% O_2_) with the nasal mask on, lambs were exposed to 10% O_2_ for 10 min. Thereafter, lambs were returned to air for a further 5 min recording. Rectal temperature and arterial blood samples were taken in order to assess pH, PaO_2_, and PaCO_2_ at the beginning and end of the 3-min baseline, as well as after 2, 6, and 10 min of hypoxia, and finally 1, 3, and 5 min after the return to normoxia.

#### Neurohistopathological studies of the brain

Following euthanasia at the end of experimental day 3 (D3), the brains of all 11 lambs were immediately perfused through the carotid arteries with cold 0.1 M phosphate buffered saline (pH 7.4) followed by 4% paraformaldehyde diluted in PBS. The brain was removed *en bloc* and post-fixed for 48 h. Selected tissue blocks were embedded in paraffin. Histopathological sections from all 11 brains were stained with hematoxylin-eosin and examined microscopically by experienced histomorphologists.

The same protocol was applied to three additional HB preterm lambs, which were euthanized at the peak of HB (D0) to assess bilirubin deposition and early brain inflammation at the acute phase of HB.

##### Immunohistochemical studies of glial cells in the nucleus tractus solitarius

The brainstems of the 11 lambs euthanized at D3 were sectioned at two-cm intervals, and blocks embedded in paraffin. The block encompassing the obex and the dorsomedial structures of the medulla immediately caudal and rostral to the area postrema was selected for special tissue processing. Series of 10 adjacent frontal sections (20 μm thick) were cut every 500 μm. Paraffin sections were mounted on slides, deparaffinized in xylene and rehydrated with washes in decreasing concentrations of ethanol. The first sections of each series were processed for hematoxylin and eosin staining for identification of structures and the four next slides were used for Iba1 (microglia activation; rabbit Wako 019-19741, 1:500) and GFAP (reactive astrocytes; mouse Sigma C9205, 1:100 000) staining.

##### Iba1 and GFAP immunochemical staining

After several washes in distilled water, sections were incubated in citric acid 10 mM (pH 6) and placed in a microwave for 11.5 min. After a 20 min wash in distilled water, sections were reacted for 30 min with an excess of peroxide (H_2_O_2_ 0.03%) to abolish endogenous peroxidase activity. After three 10 min washes in PBS, sections were immersed for 1 h 30 in PBS containing 2% bovine serum albumin and 1.5% normal goat serum to block nonspecific binding sites. Thereafter, the sections were incubated for 1 h at room temperature with the primary antibody diluted in the same blocking reagent and directed against Iba1 or GFAP. After three 10 min washes in PBS, sections were incubated for 1 h at room temperature with the appropriate secondary antibody (goat anti-rabbit or anti-mouse) conjugated with an HRP labeled polymer (EnVision+ System K4011 or K4007, respectively, Dako Canada Inc, Mississauga, ON). Sections were then washed twice and reacted for 1 (Iba1) or 2 (GFAP) min with 3,3′-diaminobenzidine (DAB+ substrate-chromogen solution, Dako kit) under visual inspection. After several washes in distilled water, sections were mounted on slides, air dried, dehydrated, cleared and coverslipped. In the present study, controls were carried out by omitting the primary antibody in the reaction. Such a procedure failed to produce any positive staining for Iba1 or GFAP.

### Data analysis

#### States of alertness and cardiorespiratory function

Standard electrophysiological and behavioral criteria were used to differentiate NREM sleep (stage N) from REM sleep (stage R) and wakefulness (Reix et al., 2004).

##### Spontaneous apneas

Spontaneous apneas were defined as at least two missed breaths. For each experimental day, the apnea index (number of apneas/hour), the time spent in apnea and the percentage of apneas coupled with desaturation or bradycardia were computed.

##### Laryngeal chemoreflexes

Analysis of LCR was performed as previously described within the first minute following each laryngeal injection (St-Hilaire et al., 2005) and included: (i) number of apneas and their total summed duration; (ii) number of bradycardias [defined by a decrease in heart rate (HR) >33% for >5 s] and their total summed duration; (iii) number of cardiac slowings (decrease in HR >33% for < 5 s); (iv) total summed duration of cardiac inhibition (total summed duration of both bradycardias and HR slowings); (v) % decrease in SpO_2_; and (vi) number of coughs (including laryngeal expiratory reflexes, which could not be discerned from coughs in our study).

##### Pulmonary chemoreflexes

Intravenous injection of capsaicin has previously been shown to trigger a biphasic response consisting of an apnea immediately followed by rapid shallow breathing (Diaz et al., 1999). Accordingly, in the first minute following IV capsaicin injection, analysis included: (i) total summed duration of respiratory and cardiac inhibition; (ii) number of bradycardias and cardiac slowings; (iii) % decrease in SpO_2_; (iv) respiratory rate (RR) during rapid shallow breathing, obtained between 30 and 60 s following the injection.

##### Hypoxia

Minute ventilation (VE), tidal volume (Vt), RR, HR as well as SpO_2_ were averaged on 20 s periods at each minute during baseline, hypoxic run, and return to normoxic condition.

#### Assessment of glial cell activation in the nucleus tractus solitarius

After completion of staining procedures, all sections were scanned (NanoZoomer-XR Digital slide scanner C12000, Hamamatsu Photonics, Boston MA, USA) for further observation with a computer and appropriate software (NDP.view2, Hamamatsu). The number of Iba1- and GFAP-positive cells was determined using a counting method similar to that detailed in previous immunohistochemical studies (Roda et al., 2004). Briefly, this method consists of densitometric and morphometric measurements in gray levels under x10 objective lens to discriminate stained particles from background in delimited regions of interest using ImageJ (developed by Wayne Rasband, NIH). Each stained particle with: (1) a size comprised between 11 and 100 consecutive pixels and (2) an optical density at least 1.5 times higher than that of the background (IJ-ISODATA thresholding method) was counted as an Iba1- or GFAP-positive cell. The investigator responsible for cell counting was blinded to the experimental conditions. Anatomical boundaries of structures retained for quantification [nucleus tractus solitarius (NTS) and area postrema (AP)] were first delimited on corresponding hematoxylin-eosin stained sections. Anatomical matching of sections across animals was respected. Bilateral counts of immunoreactive cells were performed for each structure clearly identified from at least 3 s. For the NTS, counts were performed within a 2 mm^2^ rectangular area centered either on the tractus solitarius in its intermediate part, or on the commissural subnucleus in its caudal part. The selected regions include the interstitial, lateral, and medial subnuclei of the NTS where cardiovascular but also laryngeal and some lung afferents terminate (Ciriello and Calaresu, 1981; Barraco et al., 1992), as well as ventrolateral, dorsal and commissural subnuclei where most of the bronchopulmonary afferents terminate (Kubin et al., 2006). For the AP, counts were determined on variable surface areas using manual selections adjusted to the size of this nucleus per side and section. These counts were used to calculate average numbers of immunoreactive cells per structure, section and animal.

#### Statistical analysis

Results were first averaged in each lamb and then averaged for each group. Values are expressed as means and SD. Normality was first tested using the Kolmogorov-Smirnov test. Quantitative continuous variables with normal distribution (data for spontaneous apneas as well as for LCR and pulmonary chemoreflexes with the exception of count data) were analyzed using generalized estimating equations with repeated measures, with group (HB or control) and experimental day (D0 or D3) as independent variables. The Mann–Whitney *U* test was used for quantitative continuous variables with asymmetrical distribution and for repeated measurements over time (hypoxia and blood gas results). Count data (number of apneas and coughs during LCR and number of cardiac slowings and bradycardias during LCR and pulmonary chemoreflexes) were analyzed using generalized estimating equations with repeated measures, with a Poisson distribution. Lastly, results for Iba1 and GFAP expression were analyzed using Student's *t*-test. All statistical analyses were performed with SPSS. A value of *p* < 0.05 was considered statistically significant. Furthermore, given the relatively small number of studied animals (related to both the complexity of the ovine model and the ethical constraint of keeping the number of ewes and lambs to a minimum), a statistical trend, defined as *p* < 0.1, was considered in the discussion.

## Results

The study was completed in six lambs (including 3 males) in the control group and five lambs (including 3 males) in the HB group. Accordingly, four ewes gave birth to twins, two to triplets and two to a single lamb. Five of the 16 lambs died from dystocia or neonatal respiratory distress (survival rate 69%). The mean birth weight was 2.38 (0.3) kg in the control group and 2.48 (0.5) kg in the HB group (*p* = 1).

**Figure 2:**
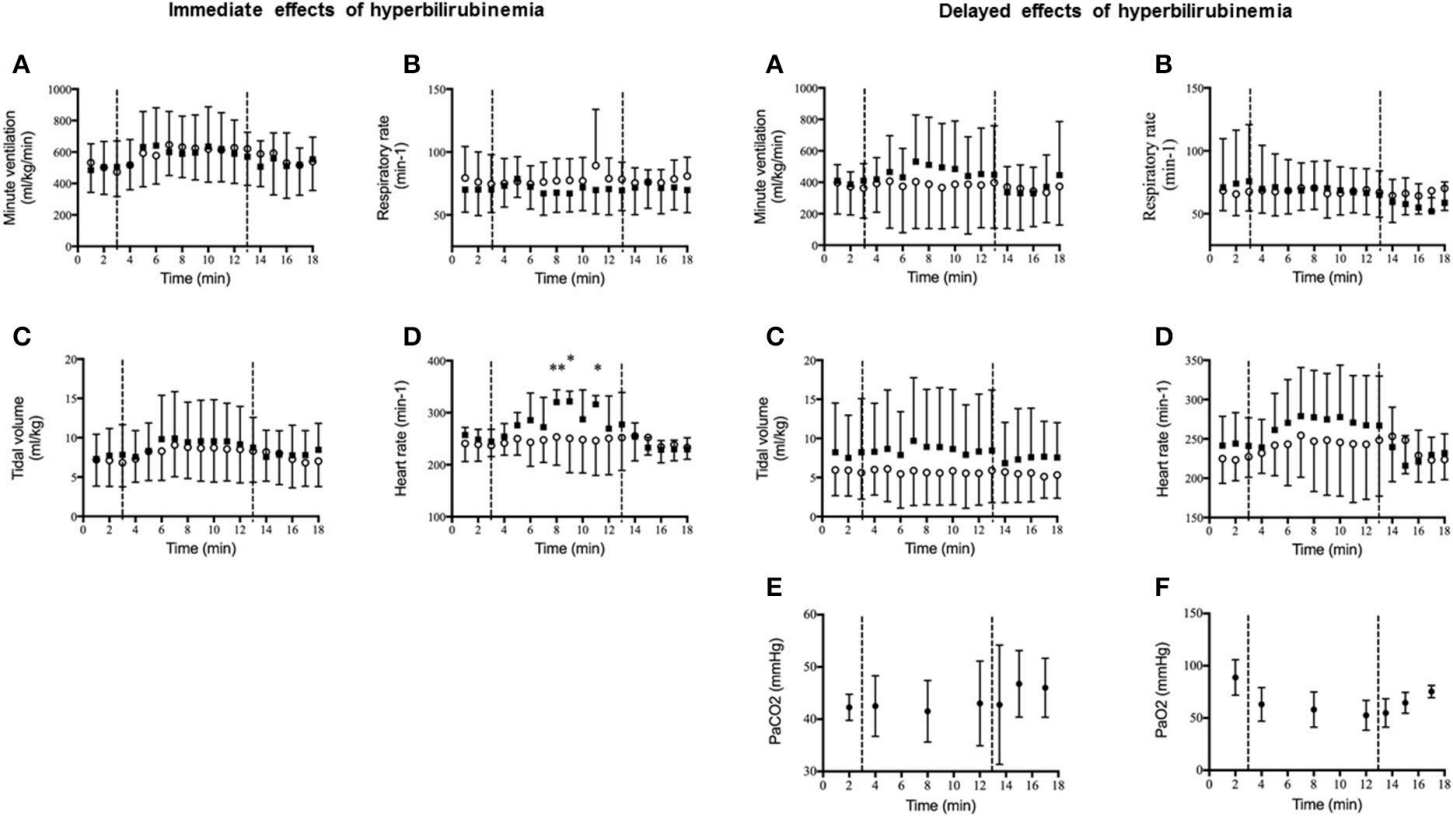
**Immediate and delayed effects of moderate hyperbilirubinemia on cardiorespiratory response to hypoxia. (A)** Minute ventilation (ml/kg/min); **(B)** respiratory rate (min^−1^); **(C)** tidal volume (ml/kg); **(D)** heart rate (min^−1^); **(E)** PaCO_2_ (mmHg); **(F)** PaO_2_ (mmHg). After a 3-min baseline recording in air, lambs were exposed to 10% O_2_ for 10 min (represented by the dashed lines), then returned to air for an additional 5-min recording. Values are reported as mean ± standard deviation. Control group (o, *n* = 6) and hyperbilirubinemia group (■, *n* = 5 immediate and *n* = 4 delayed). In order to minimize surgical intervention (surgical catheterization of the brachial artery), PaCO_2_ and PaO_2_ measurements were only obtained in 4 lambs (2 control and 2 HB lambs, and no statistical analysis were performed on these data). ^**^p < 0.05 vs. control group and ^*^p < 0.1 vs. control group.

A moderate HB was obtained for 17 h in all five HB lambs. Compared to control lambs, the “Lamb Acute Bilirubin Encephalopathy score” was significantly higher in HB (5 ± 1 vs. 0 ± 0.1) during bilirubin infusion, subsequently returning to near control levels after 24 h (1 ± 0.4) (Bourgoin-Heck et al., 2015; Figure 2C). In addition, a monotonous EEG resembling wakefulness was consistently observed in all HB lambs throughout the 7 h HB period, preventing differentiation of stage N from quiet W (Bourgoin-Heck et al., 2015; Figure 2B). Consequently, three stages of alertness were specifically defined for analysis purpose in the present study: agitated wakefulness, quiet state (= quiet wakefulness + N stage) and R stage. The latter was easily recognized in all lambs by the intense rapid eye movement activity characteristic of lambs as well as irregular breathing.

### Cardiac and respiratory function

During baseline respiration, breathing frequency of HB lambs was lower than controls on both D0 and D3 (Table 1). In addition, the time spent in apnea was significantly increased in the HB group on D0 (Table 1). No other differences were observed for baseline cardiac or respiratory activity.

**Table 1:** **Immediate and delayed effects of moderate hyperbilirubinemia on baseline cardiac and respiratory activity**.

	**Control (*n* = 5)**	**HB (*n* = 5)**	***P*-values**
**IMMEDIATE EFFECTS OF HB (EXPERIMENTAL DAY 0)**			
Ttot (s)	0.75 (0.03)	1.36 (0.09)^**^	< 0.001
Apnea index (h^−1^)	4 (2)	6 (2)	0.4
Time spent in apnea (s/h)	15 (12)	28 (9)^**^	0.03
% apnea coupled with desaturation	29 (18)	27 (24)	0.9
% apnea coupled with bradycardia	0.1 (0.1)	0.5 (0.4)	0.2
RR (ms)	270 (11)	295 (16)	0.2
**DELAYED EFFECTS OF HB (EXPERIMENTAL DAY 3)**			
Ttot (s)	1.03 (0.08)	1.37 (0.14)^**^	0.04
Apnea index (h^−1^)	4 (2)	4 (3)	0.9
Time spent in apnea (s/h)	19 (19)	21 (13)	0.8
% apnea coupled with desaturation	28 (25)	13 (11)	0.2
% apnea coupled with bradycardia	0 (0)	0.6 (0.5)	0.2
RR (ms)	284 (8)	313 (36)	0.4

Values are reported as mean (standard deviation). HB, moderate hyperbilirubinemia group; Ttot, total duration of respiratory cycle; RR, interval between successive peaks of the QRS complex.

** p < 0.05 vs. control group.

#### Laryngeal chemoreflexes

Results obtained during LCR are summarized in Table 2. Overall, on D0, moderate HB significantly blunted the cardiorespiratory inhibition normally observed during LCR, including a decrease in the number and duration of apneas, lesser cardiac inhibition and a lesser decrease in SpO_2_. At D3, however, the LCR-induced cardiorespiratory inhibition was similar between both groups (Table 2).

**Table 2:** **Immediate and delayed effects of moderate hyperbilirubinemia on laryngeal chemoreflexes**.

	**Control (*n* = 6)**	**HB (*n* = 5)**	***P*-values**
**IMMEDIATE EFFECTS OF HB (EXPERIMENTAL DAY 0)**			
Number of apneas	2 (1)	0 (0)^**^	0.01
Duration apneas (s)	7 (3)	2 (1)^*^	0.06
Duration of cardiac inhibition (s)	26 (6)	7 (3)^*^	0.07
Number of cardiac slowings	8 (2)	8 (3)	0.9
Number of bradycardias	1.5 (0.4)	0.2 (0.2)^**^	0.002
% decrease in SpO_2_	14 (3)	6 (3)^*^	0.06
Number of coughs	3 (1)	4 (3)	0.7
**DELAYED EFFECTS OF HB (EXPERIMENTAL DAY 3)**			
Number of apneas	1.2 (0.6)	0.3 (0.2)	0.1
Duration apneas (s)	8 (6)	3 (1)	0.3
Duration of cardiac inhibition (s)	15 (8)	10 (8)	0.6
Number of cardiac slowings	5.2 (1.8)	4.0 (1.4)	0.6
Number of bradycardias	0.8 (0.4)	0.6 (0.5)	0.7
% decrease in SpO_2_	6 (2)	4 (2)	0.4
Number of coughs	3 (3)	0 (0)	0.6

Values are reported as mean (standard deviation). HB, moderate hyperbilirubinemia group. Laryngeal chemoreflexes were induced by two separate injections of 2 ml of hydrochloric acid in each lamb during quiet sleep.

** p < 0.05 vs. control group and

* p < 0.1 vs. control group.

#### Pulmonary chemoreflexes

Results obtained during pulmonary chemoreflexes are summarized in Table 3. Overall, at D0, moderate HB blunted the cardiorespiratory inhibition elicited by pulmonary chemoreflexes, as shown by a decrease in total duration of respiratory and cardiac inhibition and a decrease in the number of bradycardias. The rapid and shallow breathing following the cardiorespiratory inhibition phase was similar between the two groups. At D3, however, no significant differences were noted between groups for the cardiorespiratory inhibition phase, whereas the RR during the shallow breathing phase was significantly increased in HB lambs (Table 3).

**Table 3:** **Immediate and delayed effects of moderate hyperbilirubinemia on pulmonary chemoreflexes**.

	**Control (*n* = 6)**	**HB (*n* = 4)**	***P*-values**
**IMMEDIATE EFFECTS OF HB (EXPERIMENTAL DAY 0)**			
Duration respiratory inhibition (s)	12 (1)	10 (1) ^**^	0.04
Duration cardiac inhibition (s)	13 (1)	7 (1) ^**^	< 0.001
Number cardiac slowings	1.4 (0.4)	3.7 (0.8) ^**^	0.007
Number bradycardias	1.4 (0.2)	0.5 (0.2) ^**^	0.008
% decrease SpO_2_	2 (3)	6 (4)	0.1
RR shallow breathing (cycles/min)	287 (24)	297 (13)	0.7
**DELAYED EFFECTS OF HB (EXPERIMENTAL DAY 3)**			
Duration respiratory inhibition (s)	13 (1)	12 (1)	0.7
Duration cardiac inhibition (s)	11 (2)	10 (3)	0.8
Number cardiac slowings	2.3 (0.6)	2.2 (0.4)	0.9
Number bradycardias	1 (0.2)	0.5 (0.2)	0.2
% decrease SpO_2_	0 (0.03)	0 (0.03)	0.5
RR shallow breathing (cycles/min)	231 (37)	335 (12) ^**^	0.007

Values are reported as mean (standard deviation). HB, moderate hyperbilirubinemia group; RR, respiratory rate. Pulmonary chemoreflexes were induced by two separate central IV injections of 25 μg/kg capsaicin during quiet wakefulness. One HB lamb was excluded of analysis due to technical problems

** p < 0.05 vs. control group.

#### Hypoxia

Results are illustrated in Figure 2. At D0, in response to hypoxia, both tidal volume and minute ventilation increased in all lambs, without significant difference between groups. In contrast, hypoxia increased heart rate in HB lambs only (Figure 2D, left panel). After return to normoxia, no differences were noted between the two groups. At D3, cardiorespiratory responses were not significantly different between groups (Figure 2, right panel).

### Neurohistopathological consequences of moderate hyperbilirubinemia

**Figure 3:**
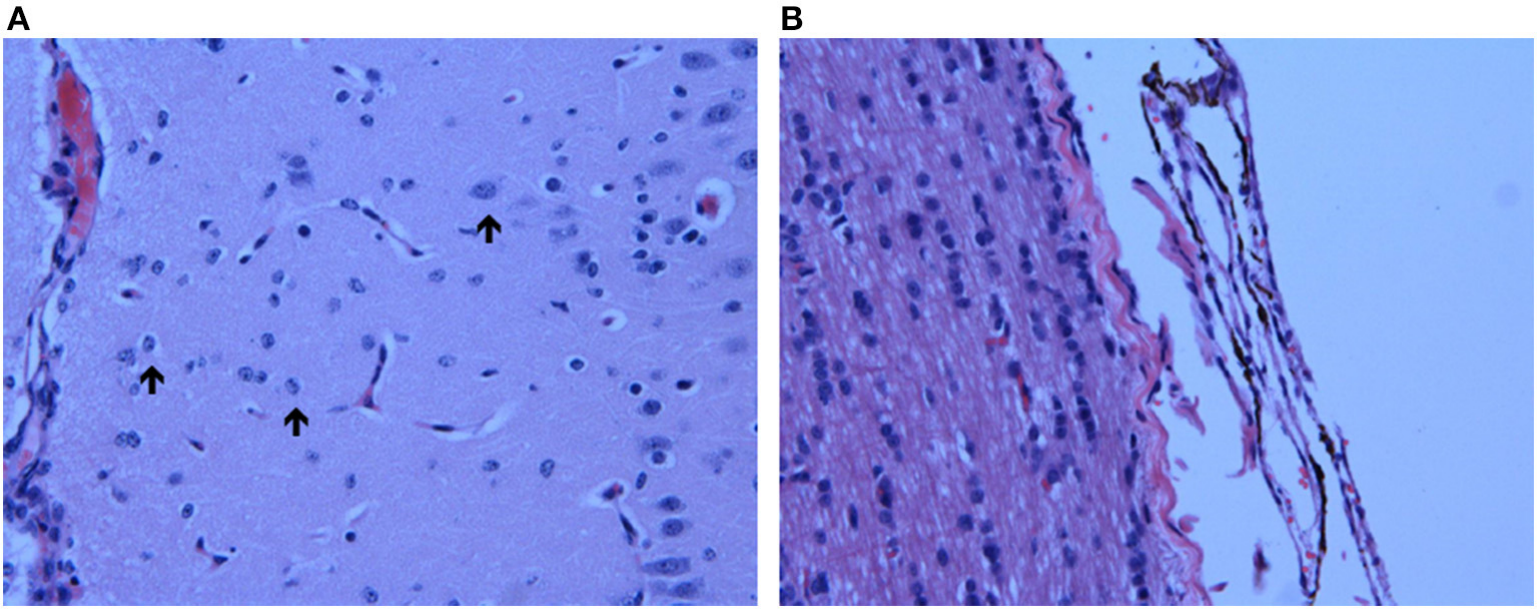
**Neuropathological Observations in HB Lambs. (A)** Presence of Alzheimer type II astrocytes, characterized by enlarged, pale nuclei with peripheral margination of chromatin (indicated by arrows), throughout the cortex of one HB preterm lamb euthanized at an HB level of 266 μmol/L; **(B)** bilirubin deposition in the pontine leptomeninges (indicated by dark brown coloration) in another HB preterm lamb with an HB level of 322 μmol/L at time of euthanasia. Images were taken at 40X.

Hematoxylin-eosin stained brain sections from the three HB animals euthanized at the peak of hyperbilirubinemia (D0) showed notable bilirubin deposits in the leptomeninges throughout the entire brain, including the brainstem (Figure 3B). While minimal bilirubin deposition was present in brain parenchyma, Alzheimer type II astrocytes [characterized by enlarged, vesicular, and at times empty-looking, nuclei with marginated chromatin and scanty cytoplasm (Ellison et al., 2013)] were noted throughout the brain, including the brainstem (Figure 3A).

Histopathological examination of the eosin-hematoxylin stained brain sections in the 11 preterm lambs euthanized at D3 showed no bilirubin deposition in any of the HB lambs. In addition, compared to controls, there were only a few foci of slight astrogliosis in the cerebral hemispheres and cerebellum in all HB lambs, as well as in the brainstem in two HB lambs.

#### Glial cell activation in the nucleus tractus solitarius

**Figure 4:**
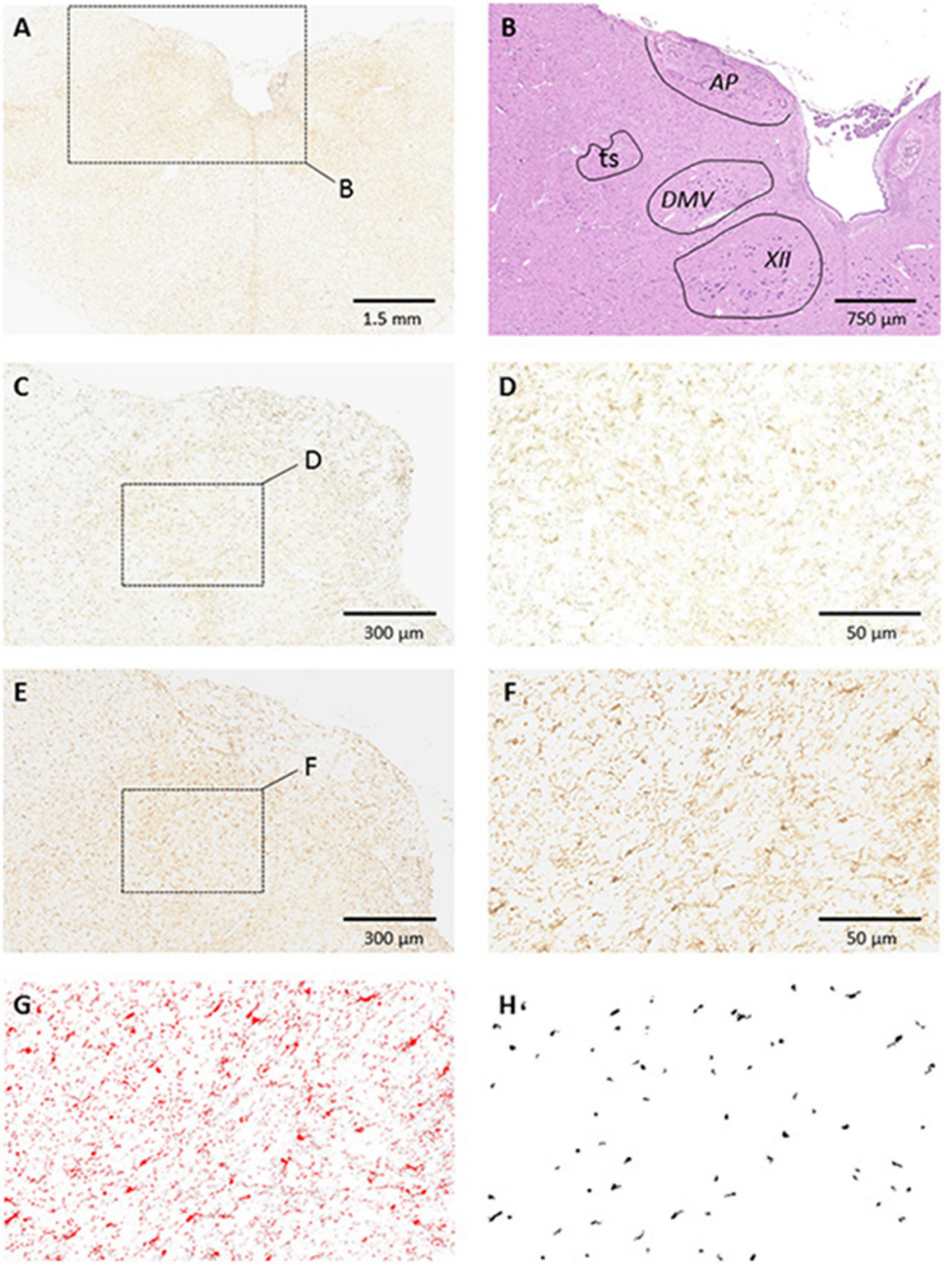
**Glial Cell Activation of Iba1 in the Dorsal Medulla. (A)** Low magnification microphotograph of a section showing Iba1 staining in the medulla. Note the higher density of Iba1 staining in the dorsomedial aspect in comparison with more ventral or lateral medullary structures; **(B)** counterstaining of the alternate section shown in **(A)** with hematoxylin-eosin illustrating the boundaries of dorsomedial nuclei; **(C,D)** example of Iba1 staining in a control animal at medium and high magnifications, respectively; **(E,F)** example of Iba1 staining in one HB preterm lamb at medium and high magnifications, respectively. Enlarged areas in **(D,F)** correspond to the medial part of the nucleus tractus solitarius (NTS). **(G,H)** Example of image processing of the section shown in **(F)**. The image was converted to 8-bit and thresholded using Image J **(G)**. Fixed morphometric and densitometric criteria were used for automatic counting of Iba1 particles **(H)**. Note the increased number of Iba1-stained particles in NTS after HB. Abbreviations: AP, area postrema; ts, tractus solitarius; DMV, dorsal motor nucleus of the vagus nerve; XII, hypoglossal motor nucleus.

Immunohistochemical staining showed that both Iba1- (Figure 4) and GFAP-positive cells were significantly increased in the NTS of HB lambs at D3 (Table 4). In addition, there was a modest increase in levels of GFAP expression in the area postrema (AP) in HB lambs when compared to controls (*p* = 0.07; Table 4). No significant difference was observed for Iba1 expression in the AP between HB and control lambs (Table 4).

**Table 4:** **Hyperbilirubinemia-induced changes in Iba1- and GFAP expression**.

	**Control**	**HB**	***P*-values**
**Iba1**			
Area postrema	188 (57), *n* = 6	207 (32), *n* = 5	0.5
Nucleus tractus solitarius	209 (20), *n* = 6	282 (36)^**^, *n* = 5	0.002
**GFAP**			
Area postrema	78 (23), *n* = 5	156 (79)^*^, *n* = 4	0.07
Nucleus tractus solitarius	35 (42), *n* = 6	111 (46)^**^, *n* = 4	0.03

Values are reported as mean (standard deviation). HB, moderate hyperbilirubinemia group.

** p < 0.05 vs. control group and

* p < 0.1 vs. control group.

## Discussion

The present study in preterm lambs suggest that sustained and moderate HB can induce acute alterations in cardiorespiratory function, including (i) a decrease in baseline respiratory rate and an increase in time spent in apneas, (ii) a blunting of the cardiorespiratory inhibition normally observed during both laryngeal and pulmonary chemoreflexes, and (iii) an increased heart rate in response to acute hypoxia. These changes were associated with an activation of Alzheimer type II astrocytes throughout the brain, including the brainstem. While most cardiovascular responses returned to normal levels 72 h after HB normalization, increased microglial and astrocyte reactivity was still observed within the nucleus tractus solitarius.

### Preterm lamb model of moderate hyperbilirubinemia

Compared to previous animal models of neonatal HB (Ahlfors et al., 1986; Brann et al., 1987; Park et al., 2001; Mesner et al., 2008; Ye et al., 2012), our preterm ovine model of moderate HB has several unique features. Indeed, in order to be clinically relevant, we induced a moderate (150–250 μmol/L) and sustained (17 h) HB in a (late) preterm animal. In contrast, previous studies reported data from more severe (from 220 to 1300 μmoles/L) and shorter duration HB (ranging from one injection to 4 h infusion) (Ahlfors et al., 1986; Brann et al., 1987; Park et al., 2001; Mesner et al., 2008; Ye et al., 2012). To our knowledge, there has been only one previous study on “moderate” HB (100–200 μmoles/L), but with considerably shorter HB duration (2 h) (Roger et al., 1995). The clinical relevance of our model is further shown by the obvious clinical manifestations (jaundice and neurological dysfunction), together with EEG signs of acute bilirubin-encephalopathy observed in all HB lambs. All the aforementioned abnormalities were reversed after bilirubin normalization, as reported in newborn infants during acute bilirubin-induced neurological dysfunction (American Academy of Pediatrics Subcommittee on Hyperbilirubinemia, 2004).

### Moderate hyperbilirubinemia and cardiorespiratory function

Observations of decreased baseline respiratory rate and increased time spent in apnea in HB lambs are in agreement with several clinical studies (Johnson et al., 2009; Amin et al., 2014; Amin and Wang, 2015). Such HB-related respiratory inhibition could potentially be mediated, at least in part, by prostaglandin E_2_ binding to its respective receptors in respiratory regions of the brainstem (Hofstetter et al., 2007).

The present results also reveal that moderate HB transiently blunted the cardiorespiratory inhibition normally observed during both laryngeal and pulmonary chemoreflexes. Such observations were somewhat unexpected, given that various postnatal stresses have been shown to increase apneas and bradycardias observed in the LCR (Lindgren et al., 1996; Frøen et al., 2000; Xia et al., 2008, 2010; St-Hilaire et al., 2010; Carreau et al., 2011). Nevertheless, the blunting of LCR observed in HB lambs in the present study may be non-beneficial, due to the heightened risk of aspiration as a result of the lack of efficient glottal closure. Unfortunately, this cannot be ascertained, for the design of the present study did not include the assessment of tracheal aspiration. The HB-related alteration in cardiorespiratory activity was further confirmed by the present observations in HB lambs, namely that (i) the cardiorespiratory inhibition normally triggered by pulmonary C-fiber stimulation was blunted, and (ii) the heart rate was increased in response to acute hypoxia. The above results expand previous data obtained with severe HB in rat pups showing an altered respiratory response to hypoxia and hypercapnia (Mesner et al., 2008).

### Bilirubin deposition and brainstem glial activation

In the three preterm lambs euthanized at the peak of HB, bilirubin deposition was observed only within the leptomeninges overlying the entire brain. This is somewhat at odds with a previous report in rat pups with severe HB levels, in which bilirubin deposition was noted in the brainstem itself (Mesner et al., 2008). Such differences may be related to the lower HB level and/or to a lower permeability of the blood brain barrier in preterm lambs compared to rat pups. In addition, acute HB was associated herein with Alzheimer type II astrocytes throughout the entire brain of the three lambs, including in the brainstem. Alzheimer type II astrocytes have been reported in various acute central nervous system injuries, such as stroke or trauma. They may represent dysfunctional astrocytes (Adams and Parker, 2011) and are thought to represent glial cells in the process of transformation into reactive astrocytes (Panickar and Norenberg, 2005). Of interest, the presence of Alzheimer type II astrocytes in the cerebral cortex (Figure 3A), indicative of brain injury, was noted in concomitance with the monotonous EEG observed in all HB lambs.

In addition to the above, astrocyte and microglial activation in the NTS were detected 3 days following acute HB in preterm lambs. Cerebral glial activation, encompassing both astrocytes and microglia, has been previously reported in response to bilirubin (reviewed in Fernandes and Brites, 2009). The present observation, to the best of our knowledge, is the first report of glial activation in the NTS in response to HB. These findings provide further evidence that even a moderate HB in preterm lambs can induce an inflammatory response in the NTS region devoted to central processing of cardiorespiratory afferent inputs (Kubin et al., 2006). Both activated microglia and astrocytes could alter neuronal function such as synaptic transmission, including that involved in cardiovascular and respiratory reflexes in the NTS (Andresen and Paton, 2011). The results herein on HB-induced alterations in laryngeal and pulmonary chemoreflexes, as well as in the cardiac response to hypoxia, are in keeping with this hypothesis. Our findings are in agreement with a previous study showing abnormal ventilatory response to CO_2_ and hypoxia in rat pups with severe HB (Mesner et al., 2008). Of note, astrocytic damage in the NTS has previously been shown to interfere with cardiovascular reflex transmission by attenuating the arterial baroreflex, the arterial chemoreflex, and the von Bezold–Jarisch cardiopulmonary reflex (Lin et al., 2013). In addition, migroglial activation in the NTS was previously implicated in the impaired cardiovascular regulation observed in diabetic rats (Rana et al., 2014). Of note, the absence of a detectable bilirubin deposition in the brain parenchyma may suggest that HB in our preterm lambs exerts its effects on glial cells via the release of certain mediators by cells forming the blood brain barrier, including astrocytes (Sofroniew, 2015).

### Limitation of the study

As stated earlier, our ovine model provides unique characteristics (preterm birth, both moderate and sustained hyperbilirubinemia), which are of particular relevance for assessing the physiological consequences of hyperbilirubinemia in human newborns. One limitation may be that, contrary to humans, preterm newborn lambs (for yet unknown reasons) do not naturally develop hyperbilirubinemia.

## Conclusion

Our results show that moderate and sustained HB alters baseline respiration and reflex cardiorespiratory function. Compared to previous reports, the novelty of our study stems from the fact that (i) results were obtained with moderate and clinically relevant HB levels, (ii) in addition to respiratory impairment, cardiac function was also shown to be altered, and (iii) glial activation was revealed in the nucleus tractus solitarius, which is a key component in the control of cardiorespiratory function.

## Author contributions

M-LS, MB-H, NS, and J-PP conceived and designed the study. M-LS and FC conceived the bilirubin solution. M-LS, MB-H and NS performed the animal experiments. M-LS, CG, MR, HK analyzed the data. M-LS, NS, CG, MR, HK, and J-PP interpreted the results obtained. NS, CG, and J-PP drafted the manuscript. M-LS, MB-H, NS, FC, CG, MR, HK, and J-PP revised the manuscript. All authors read and approved the final version of the manuscript.

## Funding

This study was supported by an operating grant from the Canadian Institutes of Health Research. J-PP is the holder of the Canada Research Chair in Neonatal Respiratory Physiology and a member of the *Centre de recherche du Centre hospitalier universitaire de Sherbrooke*. M-LS was the recipient of a Ph.D. scholarship from the Canadian Thoracic Society and the *Fonds de recherche du Québec - Nature et technologies* (FQRNT).

### Conflict of interest statement

The authors declare that the research was conducted in the absence of any commercial or financial relationships that could be construed as a potential conflict of interest.
